# Comparison of the major virulence-related genes of *Listeria monocytogenes* in Internalin A truncated strain 36-25-1 and a clinical wild-type strain

**DOI:** 10.1186/1471-2180-14-15

**Published:** 2014-01-28

**Authors:** Daisuke Kyoui, Hajime Takahashi, Satoko Miya, Takashi Kuda, Bon Kimura

**Affiliations:** 1 Department of Food Science and Technology, Faculty of Marine Science, Tokyo University of Marine Science and Technology, 4-5-7 Konan, Minato, Tokyo 108-8477, Japan

**Keywords:** *Listeria monocytogenes*, Internalin A, Next-generation-sequencing

## Abstract

**Background:**

Internalin A (InlA) facilitates the invasion of *Listeria monocytogenes* into a host cell. Some strains of *Listeria monocytogenes* express truncated forms of InlA, which reduces invasiveness. However, few virulence-related genes other than *inlA* have been analyzed in InlA-truncated strains. In the present study, we sequenced the draft genome of strain 36-25-1, an InlA-truncated strain, with pyrosequencing and compared 36 major virulence-related genes in this strain and a clinical wild-type strain.

**Results:**

Strain 36-25-1 possessed all of the virulence-related genes analyzed. Of the analyzed genes, only 4 genes (*dltA*, *gtcA*, *iap*, and *inlA*) differed when the nucleotide sequences of strain 36-25-1 and the clinical wild-type strain were compared. Analysis of the deduced amino acid sequences found no mutations that significantly influenced virulence in genes other than *inlA*.

**Conclusions:**

The virulence-associated genes in strain 36-25-1 differ little from those of the clinical wild-type strain, indicating that a slight mutation in the nucleotide sequence determines the virulence of the InlA-truncated strain. In addition, the results suggest that, aside from InlA-mediated cell invasiveness, there is almost no difference between the virulence of strain 36-25-1 and that of the clinical wild-type strain.

## Background

*Listeria monocytogenes* is a gram-positive, non-sporulating foodborne infectious pathogen. The pathogen can cause serious diseases, such as septicemia and meningitis, especially in high-risk groups (pregnant women, neonates, and immunocompromised people) with a high mortality rate of 20–30% [1]. *L. monocytogenes* is ubiquitous in nature; it can survive under conditions of high salt and low pH. Because, it can grow even at low temperatures, the bacterium can be found in many kinds of foods during storage [2]. In particular, ready-to-eat (RTE) foods, which do not require heat cooking, are a main source of foodborne listeriosis cases [3-5].

Internalin A (InlA) plays an important role in *L. monocytogenes* invasion by attaching to host cells [6-9]. However, some *L. monocytogenes* strains express a truncated form of InlA, which lowers the invasion rate [10-14]. Truncated InlA, caused by a premature stop codon (PMSC) in *inlA*, often lacks the LPXTG motif that anchors InlA to the surface of the pathogen, leading to a decrease in invasiveness [12,13]. Some reports have shown that InlA-truncated strains account for 35–45% of *L. monocytogenes* in RTE foods [15,16]. However, aside from invasiveness, the virulence of these mutated strains has not been studied.

Our previous study showed that an InlA-truncated strain had wild-type PrfA, which regulates the expression of virulence related genes [11]. On the other hand, Tèmoin et al. (2008) reported that all of 5 InlA-truncated strains analyzed had the same amino acid sequence mutations in the migration factor *plcA* and the invasion factor *inlB*[17]. However, approximately 50 genes are related to the *L. monocytogenes* infection cycle [18], and most of them have not been investigated in strains with truncated InlA. A small number of studies have investigated virulence-related genes in InlA-truncated strains [11,17]; the studies do not completely explain the virulence of the strains.

This study aimed to identify the major virulence-related gene sequences present in InlA-truncated strains. In recent years, the analysis of bacterial whole genomes has become faster and easier with the development of next-generation sequencing methods such as pyrosequencing. In this study, we used pyrosequencing to construct a draft sequence of strain 36-25-1, and we compared 36 main virulence-related genes in the InlA-truncated strain and a clinical wild-type strain.

## Results

### Presence of virulence-related genes

After *de novo* assembly of the reads for strain 36-25-1, the total contig length was 2,957,538 bp with a peak depth of 11.0. The contigs aligned to 2,861,194 bp of the EGDe whole genome sequence, and showed 99.84% identity (Table 1).

**Table 1:** The draft sequences results

	**36-25-1**	**EGDe**
Total bases (bp)	2,957,538 (11.0*)	2,944,528
Alignment length (bp)	2,861,194	
Similarity	99.84 %	

*The number in the parentheses shows the peak depth of *de novo* assembly results.

In strain 36-25-1, 36 open reading frames (ORFs) showed high similarity with the 36 EGDe virulence-related genes, indicating that strain 36-25-1 has all of these genes.

### Comparison of the nucleotide and amino acid sequences of the virulence-related genes

**Figure 1:**
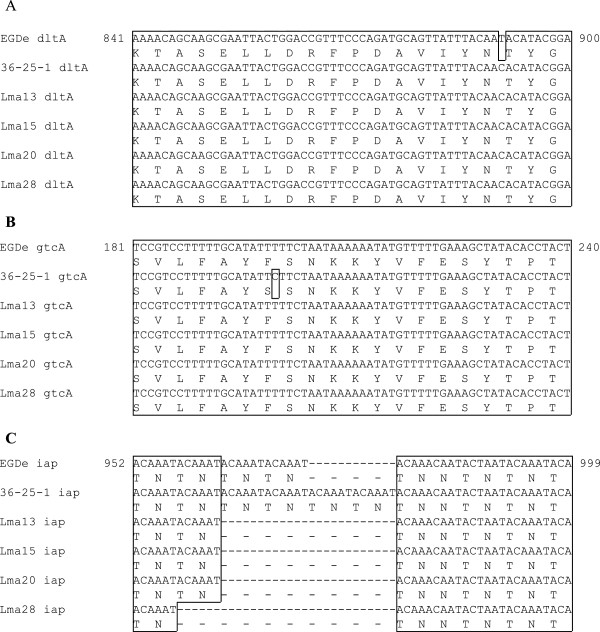
**The alignment of mutation loci in EGDe and InlA-truncated strains.** Nucleotide sequences and amino acid sequences are shown for each strain. The numbers shown on the both sides mean the nucleotide sequence positions in the ORF of strain EGDe. The frames show identical sequences among the strains. **(A)** The alignment *dltA*. **(B)** The alignment *gtcA*. **(C)** The alignment *iap*.

Nucleotide mutations were found in 4 genes (*dltA, gtcA, inlA,*and *iap*) of strain 36-25-1, when compared to EGDe (Table 2). Substitutions of 1 bp were found in *dltA*, *gtcA*, and *inlA*. The mutation in *iap* was an insertion of 12 bp (Figure 1).

**Table 2:** The alignment results of 36-25-1 and EGDe

**Gene**	**Mutation type**	**Mutation loci**	EGDe allelic type^*^	36-25-1 allelic type^*^	**Function of mutation loci**
*actA*	N/A				
*ami*	N/A				
*aut*	N/A				
*ctaP*	N/A				
*dltA*	Silence	891	T (N)	C (N)	AMP binding site
*fbpA*	N/A				
*fri*	N/A				
*gap*	N/A				
*gtcA*	Missense	200	T (F)	C (S)	Function unknown
*inlA*	Nonsense	1578	A (K)	T (*)	Listeria-bacteroides repeat domain
*inlB*	N/A				
*inlC*	N/A				
*inlH*	N/A				
*inlJ*	N/A				
*lap*	N/A				
*lgt*	N/A				
*hly*	N/A				
*lntA*	N/A				
*lpeA*	N/A				
*lplA1*	N/A				
*lsp*	N/A				
*mpl*	N/A				
*mprF*	N/A				
*murA*	N/A				
*oppA*	N/A				
*iap*	Insertion	982	N/A	ACAAATACAAAT (TNTN)	Non coding region
*pgdA*	N/A				
*pgl*	N/A				
*plcA*	N/A				
*plcB*	N/A				
*prsA2*	N/A				
*pycA*	N/A				
*recA*	N/A				
*sipZ*	N/A				
*sod*	N/A				
*svpA*	N/A				

^*^In the allelic type columns, the amino acid residues are described in the parenthesis.

In *dltA*, thymine was changed to cytosine at position 891 of the ORF. This mutation is a silent mutation, which does not cause an amino acid sequence change (Figure 1A). On the other hand, the mutation in *gtcA* is a missense mutation, which affects the amino acid sequence; substitution of thymine with cytosine position 200 changed the phenylalanine in EGDe to serine in strain 36-25-1 (Figure 1B). The inserted region in *iap* is a tandem repeat sequence. Whereas EGDe has 5 repeats of the ACAAAT motif, strain 36-25-1 has 7 repeats, resulting in 2 additional threonine-asparagine (TN) repeats (Figure 1C). Among the genes analyzed, a nonsense mutation was found only in *inlA* (Additional file 1).

### Mutation of virulence-related genes in other InlA-truncated strains

The 4 genes, in which the nucleotide sequences differed between strain 36-25-1 and EGDe were also sequenced in other InlA-truncated strains (Lma13, Lma15, Lma20, and Lma28). The silent mutation in *dltA* was found in all of the InlA-truncated strains (Figure 1A). On the other hand, the missense mutation in *gtcA* found in 36-25-1 was not found in the other InlA-truncated strains (Figure 1B). As for the tandem repeat of the ACAAAT motif in *iap*, strains Lma13, Lma15, and Lma20 had 3 repeats; strain Lma28 had 2 repeats (Figure 1C).

## Discussion

### Virulence-related genes in strain 36-25-1

The contig of the 36-25-1 strain constructed by *de novo* assembly showed similarity as high as 99.84% in the regions that aligned with the EGDe strain. In addition, this strain possessed all of the 36 virulence-associated genes analyzed. The genus *Listeria* is considered to have lost virulence-associated genes as it differentiated from ancestors that showed virulence [19]. Multiple virulence-associated genes are missing in strain 4a, a serotype of *L. monocytogenes* showing no virulence [20]. Because strain 36-25-1 possesses all of the 36 genes investigated in the present study, we conclude that the InlA-truncated strain has not undergone changes that have resulted in any major loss of regions present in the clinical wild-type strain.

### Virulence-related genes with mutations

Among the 36 virulence-associated genes in strain 36-25-1, 32 genes possess a sequence identical to that of the corresponding gene in the EGDe strain. Therefore, we conclude that the virulence of these genes is the same in the 36-25-1 and EGDe strains. Nucleotide sequence differences were found in only 4 genes (*dltA*, *gtcA*, *iap*, and *inlA*).

*dltA* is a part of the *dlt* operon, which is composed of 4 genes that function in the addition of alanine to lipoteichoic acid (LTA) [21]. Experiments using a strain in which *dltA* was artificially inactivated suggest that *dltA* influences the electric charge of the bacterial surface to increase adhesiveness to host cells [22]. The *dltA* mutation found in strain 36-25-1 is present in all other InlA-truncated strains examined in this study. Whether or not this mutation is characteristic of InlA-truncated strains requires investigation of other clinical wild-type strains. However, this mutation does not influence the phenotype of these strains as a silent mutation.

Similar to *dltA*, *gtcA* is involved in the addition of a saccharide to LTA [21]. In the present study, the nucleotide sequence of *gtcA* in strain 36-25-1 differed from that in the EGDe strain, and the encoded amino acid sequence differed as well. However, this mutation is not common to InlA-truncated strains: the mutation was not found in the other InlA-truncated strains examined.

The mutation in *iap* is in the tandem repeat region, in which the number of repeats has been reported to vary even among clinical wild-type strains [23-26]. p60, encoded by *iap*, is involved in the movement of *L. monocytogenes* inside a host cell and in cell-to-cell propagation [24]. p60 possesses multiple LysM motifs at its C-terminus, which are used to bind to the cell wall of *L. monocytogenes*, and an SH3 structure at the N-terminus exposed outside the cell [27,28]. The *iap* mutation in strain 36-25-1 occurred between the LysM domain and the SH3 structure. Therefore, we conclude that this mutation has almost no influence on the functions of these domains.

### Pathogenicity of InlA truncated strain

The results of the present study indicate that, except for InlA-mediated cell invasiveness, virulence in the InlA-truncated 36-25-1 strain is almost equivalent to that in a clinical wild-type strain. However, these results differ from those of a study by Témoin *et al*. (2008), which demonstrated that 3 virulence genes, including *inlA*, were simultaneously truncated [17]. In addition, Van Stelten *et al*. (2011), after orally administering an InlA-truncated strain to guinea pigs, reported that only the translocation rate to the spleen was lower in the truncated strain, when compared to a clinical wild-type strain [14]. Moreover, in the study by Olier *et al*. (2005) using a strain that originally showed virulence but was genetically modified to express truncated InlA, the mortality of chicken embryos infected with the transformed strain was lower than that of chickens infected with a clinical wild-type strain [13]. These reports indicate that aspects of virulence other than cell invasiveness differ between InlA-truncated strains and clinical wild-type strains; this cannot be explained by the results of the present study. Therefore, we expect that mutations in the virulence-associated genes of InlA-truncated strains will exhibit heterogeneity. The results from InlA-truncated strains other than strain 36-25-1 in the present study support this hypothesis. In addition, many *L. monocytogenes* genes have functions that are not yet elucidated. Hence, we cannot exclude the possibility that genes not analyzed in the present study contribute to the differences between InlA-truncated strains and clinical wild-type strains. Analyzing other InlA-truncated strains and determining the unknown functions of *L. monocytogenes* genes will resolve these questions.

## Conclusions

In the present study, we analyzed the major virulence-associated genes in strain 36-25-1, an InlA-truncated strain. With the exception of *inlA*, the virulence-associated genes in the InlA-truncated strain are almost identical to those in a clinical wild-type strain. The results indicate that a slight mutation in the nucleotide sequence, such as a PMSC, determines the virulence of InlA-truncated strains. In addition, post-translational analysis of each gene indicated that, except for InlA-mediated cell invasiveness, the virulence of the 36-25-1 strain is equivalent to that of clinical wild-type strains. However, this result does not completely explain the results of previous studies on InlA-truncated strains. To clarify the relationship between virulence and genes in InlA-truncated strains, analyses of more InlA-truncated strains, including pyrosequencing, and studies of gene expression are required to understand the virulence of InlA-truncated strains deeper.

## Methods

### Bacterial strains used in this study

*L. monocytogenes* strain 36-25-1, with truncated InlA, was sequenced by whole genome shot gun sequencing to analyze virulence-related genes. The low invasiveness of the strain compared to that of the wild-type strain was shown in our previous study [11].

In addition, four InlA-truncated strains (Lma13, Lma15, Lma20, and Lma28) isolated from raw meat products were sequenced by Sanger sequencing for reference [29].

The whole genome sequence of EGDe, a clinical wild-type strain, was obtained from GenBank (GenBank accession no. NC 003210).

### Genome extraction

All *L. monocytogenes* strains were cultured overnight in brain heart infusion broth (Eiken Chemical, Tokyo, Japan) at 37°C. The bacterial DNA was extracted using the phenol-chloroform and ethanol precipitation method [30]. One milliliter of enriched culture was centrifuged at 10,000 × *g* for 10 min, and bacterial cells were incubated in 567 μL of Tris-EDTA buffer containing lysozyme (2 mg/mL) for 1 h at 37°C. Cells were lysed by the addition of 30 μL of 10% (wt/vol) sodium dodecyl sulfate and 3 mL of 20 mg/mL proteinase K, with incubation for 1 h at 37°C. Next, 100 μL of 5 M NaCl was added, and DNA was extracted with chloroform–isoamyl alcohol (24:1) followed by phenol–chloroform–isoamyl alcohol (25:24:1). DNA was then precipitated with isopropanol, washed with 70% ethanol, and dried. Purified DNA was dissolved in Tris-EDTA buffer and used as the DNA template for whole genome shot gun sequencing and Sanger sequencing.

### Whole genome shot gun sequencing and *de novo* assembly

For whole genome shot gun sequencing, a Roche GS Junior platform (Roche, Basel, Schweiz) was employed using a GS Junior Rapid Library Preparation kit and GS Junior emPCR kit (Lib-L) according to the manufacture’s protocol. The read sequences were used to construct a contig without a reference sequence by *de novo* assembly using the GS *De Novo* Assembler (Roche, Basel, Schweiz). In this assembly, the program parameters were set to: seed step, 12; seed length, 16; seed count, 1; minimum overlap, 10; and minimum identity, 90.

### Extraction of virulence-related gene loci and comparison analysis

The contigs of strain 36-25-1 and the EGDe whole genome sequence were aligned using NUCmer, an application of MUMmer 3.0 (http://mummer.sourceforge.net/).

The virulence-related gene loci of strain 36-25-1 were extracted from the contigs using GenomeTraveler (In Silico Biology, Kanagawa, Japan). Briefly, among the ORFs extracted from the contigs, those that showed high identity with EGDe virulence-related genes were selected for further analysis.

The extracted gene sequences were aligned with the EGDe sequences by GENETYX ver11.0.0 (Genetyx, Tokyo, Japan) to identify nucleotide mutations. When a genomic mutation was found, the corresponding amino acid sequences were also compared.

### Sanger sequencing at the mutation loci

When gene mutations were identified in strain 36-25-1, Sanger sequencing was conducted for confirmation. Other InlA-truncated strains (Lma13, Lma15, Lma20, and Lma28) were sequenced at the same loci. The primers for PCR amplification and Sanger sequencing were designed using ABI PRISM Primer Express v2.0.0 (Life Technologies, Carlsbad, CA, USA) (Table 3). The PCR reaction mixture, prepared in 50 μL, contained 10 mM Tris–HCl (pH 8.3), 50 mM KCl, 1.5 mM MgCl_2_, 50 pmol of each primer, 2.5 mM of each dNTP, 25 ng template DNA, and 1.25 U Takara *Taq* DNA polymerase (Takara Bio, Shiga, Japan). The PCR program consisted of 94°C for 4 min; 30 cycles of 94°C for 30 sec, annealing temperature for 30 sec, and 72°C 2 min; and 72°C for 4 min (Table 3). The PCR products were purified using Agencourt AMPure (Beckman Coulter, Brea, CA, USA). Sanger sequencing was conducted using an Applied Biosystems 3130 Genetic Analyzer (Life Technologies, Carlsbad, CA, USA) using a Big-Dye Terminator ver3.1 Cycle Sequencing Kit (Life Technologies, Carlsbad, CA, USA) and Agencourt CleanSEQ (Beckman Coulter, Brea, CA, USA), according to each manufacturer’s protocol.

**Table 3:** The primers used in PCR amplification and sequencing for confirmation of the mutation

**Gene**	**Forward**	**Reverse**	**Annealing temperature**
*dltA*	AAGTAGTGCAGTTTAGGAGAGGA	AGATTGTACCACCGGATGTC	58.0
*gtcA*	TTGAGCTCTTAGTAGAACCTGAC	CTGGTTTCGCTATCTCATTAG	54.5
*iap*	CAAAATGCTACTACACACGCT	GTCAAAGAATACTAAATCACCAGC	56.5

## Availability of supporting data

The draft genome sequences of *L. monocytogenes* strain 36-25-1 are available in DDBJ/EMBL/GenBank under accession number BASN01000001-BASN01000122. The gene sequences of other strains Lma13, Lma15, Lma20 and Lma28 are available under accession number AB845328-845343.

## Competing interests

The authors declare that they have no competing interests.

## Authors’ contributions

Conception and design of this study: HT, KB. Laboratory work and data analysis: DK, HT. Manuscript writing, review and revision: DK, HT, SM, TK. All authors read and approved the final manuscript.

## Supplementary Material

Additional file 1 **The alignment of *inlA *** **in EGDe and InlA truncated strains.** Nucleotide sequences and amino acid sequences are shown for each strain. The numbers shown on the both sides mean the nucleotide sequence positions in the ORF of strain EGDe. The frames show identical sequences among the strains. Click here for file
