# Supplementary material for: Comparison of the major virulence-related genes of Listeria monocytogenes in Internalin A truncated strain 36-25-1 and a clinical wild-type strain
Source: BMC Microbiol. 2014 Jan 28;14:15. doi: 10.1186/1471-2180-14-15 (PMC3917698; doi:10.1186/1471-2180-14-15)
Supplement: Additional file 1 — The alignment of inlA in EGDe and InlA truncated strains. Nucleotide sequences and amino acid sequences are shown for each strain. The numbers shown on the both sides mean the nucleotide sequence positions in the ORF of strain EGDe. The frames show identical sequences among the strains. [file 1471-2180-14-15-S1.pdf]

```
EGDe inlA       1   GTGAGAAAAAAACGATATGTATGGTTGAAAAGTATACTAGTAGCAATATTAGTATTTGGC  60
                    V  R  K  K  R  Y  V  W  L  K  S  I  L  V  A  I  L  V  F  G
36-25-1 inlA        GTGAGAAAAAAACGATATGTATGGTTGAAAAGTATACTAGTAGCAATATTAGTATTTGGC
                    V  R  K  K  R  Y  V  W  L  K  S  I  L  V  A  I  L  V  F  G
Lma10 inlA          GTGAGAAAAAAACGATATGTATGGTTGAAAAGTATACTAGTAGCAATATTAGTATTTGGC
                    V  R  K  K  R  Y  V  W  L  K  S  I  L  V  A  I  L  V  F  G
Lma13 inlA          GTGAGAAAAAAACGATATGTATGGTTGAAAAGTATACTAGTAGCAATATTAGTATTTGGC
                    V  R  K  K  R  Y  V  W  L  K  S  I  L  V  A  I  L  V  F  G
Lma20 inlA          GTGAGAAAAAAACGATATGTATGGTTGAAAAGTATACTAGTAGCAATATTAGTATTTGGC
                    V  R  K  K  R  Y  V  W  L  K  S  I  L  V  A  I  L  V  F  G
Lma28 inlA          GTGAGAAAAAAACGATATGTATGGTTGAAAAGTATACTAGTAGCAATATTAGTATTTGGC
                    V  R  K  K  R  Y  V  W  L  K  S  I  L  V  A  I  L  V  F  G

EGDe inlA      61   AGCGGAGTATGGATTAACACGAGTAACGGGACAAATGCTCAGGCAGCTACAATTACACAA  120
                    S  G  V  W  I  N  T  S  N  G  T  N  A  Q  A  A  T  I  T  Q
36-25-1 inlA        AGCGGAGTATGGATTAACACGAGTAACGGGACAAATGCTCAGGCAGCTACAATTACACAA
                    S  G  V  W  I  N  T  S  N  G  T  N  A  Q  A  A  T  I  T  Q
Lma10 inlA          AGCGGAGTATGGATTAACACGAGTAACGGGACAAATGCTCAGGCAGCTACAATTACACAA
                    S  G  V  W  I  N  T  S  N  G  T  N  A  Q  A  A  T  I  T  Q
Lma13 inlA          AGCGGAGTATGGATTAACACGAGTAACGGGACAAATGCTCAGGCAGCTACAATTACACAA
                    S  G  V  W  I  N  T  S  N  G  T  N  A  Q  A  A  T  I  T  Q
Lma20 inlA          AGCGGAGTATGGATTAACACGAGTAACGGGACAAATGCTCAGGCAGCTACAATTACACAA
                    S  G  V  W  I  N  T  S  N  G  T  N  A  Q  A  A  T  I  T  Q
Lma28 inlA          AGTGGAGTATGGATTAACACGAGCAACGGGACAAATGCTCAGGCAGCTACAATTACACAA
                    S  G  V  W  I  N  T  S  N  G  T  N  A  Q  A  A  T  I  T  Q

EGDe inlA     121   GATACTCCTATTAATCAGATTTTTACAGATACAGCTCTAGCGGAAAAAATGAAGACGGTC  180
                    D  T  P  I  N  Q  I  F  T  D  T  A  L  A  E  K  M  K  T  V
36-25-1 inlA        GATACTCCTATTAATCAGATTTTTACAGATACAGCTCTAGCGGAAAAAATGAAGACGGTC
                    D  T  P  I  N  Q  I  F  T  D  T  A  L  A  E  K  M  K  T  V
Lma10 inlA          GATACTCCTATTAATCAGATTTTTACAGATACAGCTCTAGCGGAAAAAATGAAGACGGTC
                    D  T  P  I  N  Q  I  F  T  D  T  A  L  A  E  K  M  K  T  V
Lma13 inlA          GATACTCCTATTAATCAGATTTTTACAGATACAGCTCTAGCGGAAAAAATGAAGACGGTC
                    D  T  P  I  N  Q  I  F  T  D  T  A  L  A  E  K  M  K  T  V
Lma20 inlA          GATACTCCTATTAATCAGATTTTTACAGATACAGCTCTAGCGGAAAAAATGAAGACGGTC
                    D  T  P  I  N  Q  I  F  T  D  T  A  L  A  E  K  M  K  T  V
Lma28 inlA          GATACTCCTATTAATCAGATTTTTACAGACGCAGCTCTAGCGGAAAAAATGAAGACGGTC
                    D  T  P  I  N  Q  I  F  T  D  A  A  L  A  E  K  M  K  T  V

EGDe inlA     181   TTAGGAAAAACGAATGTAACAGACACGGTCTCACAAACAGATCTAGACCAAGTTACAACG  240
                    L  G  K  T  N  V  T  D  T  V  S  Q  T  D  L  D  Q  V  T  T
36-25-1 inlA        TTAGGAAAAACGAATGTAACAGACACGGTCTCACAAACAGATCTAGACCAAGTTACAACG
                    L  G  K  T  N  V  T  D  T  V  S  Q  T  D  L  D  Q  V  T  T
Lma10 inlA          TTAGGAAAAACGAATGTAACAGACACGGTCTCACAAACAGATCTAGACCAAGTTACAACG
                    L  G  K  T  N  V  T  D  T  V  S  Q  T  D  L  D  Q  V  T  T
Lma13 inlA          TTAGGAAAAACGAATGTAACAGACACGGTCTCACAAACAGATCTAGACCAAGTTACAACG
                    L  G  K  T  N  V  T  D  T  V  S  Q  T  D  L  D  Q  V  T  T
Lma20 inlA          TTAGGAAAAACGAATGTAACAGACACGGTCTCACAAACAGATCTAGACCAAGTTACAACG
                    L  G  K  T  N  V  T  D  T  V  S  Q  T  D  L  D  Q  V  T  T
Lma28 inlA          TTAGGAAAAACGAATGTAACAGACACGGTCTCACAAACAGATCTAGACCAAGTTACGACG
                    L  G  K  T  N  V  T  D  T  V  S  Q  T  D  L  D  Q  V  T  T
```

```
EGDe inlA      241 CTTCAGGCGGATAGATTAGGGATAAAATCTATCGATGGAGTGGAATACTTGAACAATTTA 300
                   L  Q  A  D  R  L  G  I  K  S  I  D  G  V  E  Y  L  N  N  L
36-25-1 inlA       CTTCAGGCGGATAGATTAGGGATAAAATCTATCGATGGAGTGGAATACTTGAACAATTTA
                   L  Q  A  D  R  L  G  I  K  S  I  D  G  V  E  Y  L  N  N  L
Lma10 inlA         CTTCAGGCGGATAGGTTAGGGATAAAATCTATCGATGGAGTGGAATACTTGAACAATTTA
                   L  Q  A  D  R  L  G  I  K  S  I  D  G  V  E  Y  L  N  N  L
Lma13 inlA         CTTCAGGCGGATAGGTTAGGGATAAAATCTATCGATGGAGTGGAATACTTGAACAATTTA
                   L  Q  A  D  R  L  G  I  K  S  I  D  G  V  E  Y  L  N  N  L
Lma20 inlA         CTTCAGGCGGATAGGTTAGGGATAAAATCTATCGATGGAGTGGAATACTTGAACAATTTA
                   L  Q  A  D  R  L  G  I  K  S  I  D  G  V  E  Y  L  N  N  L
Lma28 inlA         CTTCAGGCGGATAGGTTAGGGATAAAATCTATCGATGGAGTGGAATACTTGAACAATTTA
                   L  Q  A  D  R  L  G  I  K  S  I  D  G  V  E  Y  L  N  N  L

EGDe inlA      301 ACACAAATAAATTTCAGCAATAATCAACTTACGGACATAACGCCACTTAAAAATTTAACT 360
                   T  Q  I  N  F  S  N  N  Q  L  T  D  I  T  P  L  K  N  L  T
36-25-1 inlA       ACACAAATAAATTTCAGCAATAATCAACTTACGGACATAACGCCACTTAAAAATTTAACT
                   T  Q  I  N  F  S  N  N  Q  L  T  D  I  T  P  L  K  N  L  T
Lma10 inlA         ACACAAATAAATTTCAGCAATAATCAACTTACGGACATAACGCCACTTAAAAATTTAACT
                   T  Q  I  N  F  S  N  N  Q  L  T  D  I  T  P  L  K  N  L  T
Lma13 inlA         ACACAAATAAATTTCAGCAATAATCAACTTACGGACATAACGCCACTTAAAAATTTAACT
                   T  Q  I  N  F  S  N  N  Q  L  T  D  I  T  P  L  K  N  L  T
Lma20 inlA         ACACAAATAAATTTCAGCAATAATCAACTTACGGACATAACGCCACTTAAAAATTTAACT
                   T  Q  I  N  F  S  N  N  Q  L  T  D  I  T  P  L  K  N  L  T
Lma28 inlA         ACACAAATAAATTTCAGCAATAATCAACTTACGGACATAACGCCACTTAAAAATTTAACT
                   T  Q  I  N  F  S  N  N  Q  L  T  D  I  T  P  L  K  N  L  T

EGDe inlA      361 AAGTTAGTTGATATTTTGATGAATAATAATCAAATAGCAGATATAACTCCGCTAGCTAAT 420
                   K  L  V  D  I  L  M  N  N  N  Q  I  A  D  I  T  P  L  A  N
36-25-1 inlA       AAGTTAGTTGATATTTTGATGAATAATAATCAAATAGCAGATATAACTCCGCTAGCTAAT
                   K  L  V  D  I  L  M  N  N  N  Q  I  A  D  I  T  P  L  A  N
Lma10 inlA         AAGTTAGTTGATATTTTGATGAATAATAATCAAATAGCAGATATAACTCCGCTAGCTAAT
                   K  L  V  D  I  L  M  N  N  N  Q  I  A  D  I  T  P  L  A  N
Lma13 inlA         AAGTTAGTTGATATTTTGATGAATAATAATCAAATAGCAGATATAACTCCGCTAGCTAAT
                   K  L  V  D  I  L  M  N  N  N  Q  I  A  D  I  T  P  L  A  N
Lma20 inlA         AAGTTAGTTGATATTTTGATGAATAATAATCAAATAGCAGATATAACTCCGCTAGCTAAT
                   K  L  V  D  I  L  M  N  N  N  Q  I  A  D  I  T  P  L  A  N
Lma28 inlA         AAGTTAGTTGATATTTTGATGAATAATAATCAAATAGCAGATATAACTCCGCTAGCTAAT
                   K  L  V  D  I  L  M  N  N  N  Q  I  A  D  I  T  P  L  A  N

EGDe inlA      421 TTGACGAATCTAACTGGTTTGACTTTGTTCAACAATCAGATAACGGATATAGACCCGCTT 480
                   L  T  N  L  T  G  L  T  L  F  N  N  Q  I  T  D  I  D  P  L
36-25-1 inlA       TTGACGAATCTAACTGGTTTGACTTTGTTCAACAATCAGATAACGGATATAGACCCGCTT
                   L  T  N  L  T  G  L  T  L  F  N  N  Q  I  T  D  I  D  P  L
Lma10 inlA         TTGACGAATCTAACTGGTTTGACTTTGTTCAACAATCAGATAACGGATATAGACCCGCTT
                   L  T  N  L  T  G  L  T  L  F  N  N  Q  I  T  D  I  D  P  L
Lma13 inlA         TTGACGAATCTAACTGGTTTGACTTTGTTCAACAATCAGATAACGGATATAGACCCGCTT
                   L  T  N  L  T  G  L  T  L  F  N  N  Q  I  T  D  I  D  P  L
Lma20 inlA         TTGACGAATCTAACTGGTTTGACTTTGTTCAACAATCAGATAACGGATATAGACCCGCTT
                   L  T  N  L  T  G  L  T  L  F  N  N  Q  I  T  D  I  D  P  L
Lma28 inlA         TTGACGAATCTAACTGGTTTGACTTTGTTCAACAATCAGATAACGGATCTAGACCCGCTT
                   L  T  N  L  T  G  L  T  L  F  N  N  Q  I  T  D  L  D  P  L
```

```
EGDe inlA      481 AAAAATCTAACAAATTTAAATCGGCTAGAACTATCCAGTAACACGATTAGTGATATTAGT 540
                   K  N  L  T  N  L  N  R  L  E  L  S  S  N  T  I  S  D  I  S
36-25-1 inlA       AAAAATCTAACAAATTTAAATCGGCTAGAACTATCCAGTAACACGATTAGTGATATTAGT
                   K  N  L  T  N  L  N  R  L  E  L  S  S  N  T  I  S  D  I  S
Lma10 inlA         AAAAATCTAACAAATTTAAATCGGCTAGAACTATCCAGTAACACGATTAGTGATATTAGT
                   K  N  L  T  N  L  N  R  L  E  L  S  S  N  T  I  S  D  I  S
Lma13 inlA         AAAAATCTAACAAATTTAAATCGGCTAGAACTATCCAGTAACACGATTAGTGATATTAGT
                   K  N  L  T  N  L  N  R  L  E  L  S  S  N  T  I  S  D  I  S
Lma20 inlA         AAAAATCTAACAAATTTAAATCGGCTAGAACTATCCAGTAACACGATTAGTGATATTAGT
                   K  N  L  T  N  L  N  R  L  E  L  S  S  N  T  I  S  D  I  S
Lma28 inlA         AAAAATCTAACAAATTTAAATCGGCTAGAACTATCTAGTAACACGATTAGTGATATTAGT
                   K  N  L  T  N  L  N  R  L  E  L  S  S  N  T  I  S  D  I  S

EGDe inlA      541 GCGCTTTCAGGTTTAACTAGTCTACAGCAATTATCTTTTGGTAATCAAGTGACAGATTTA 600
                   A  L  S  G  L  T  S  L  Q  Q  L  S  F  G  N  Q  V  T  D  L
36-25-1 inlA       GCGCTTTCAGGTTTAACTAGTCTACAGCAATTATCTTTTGGTAATCAAGTGACAGATTTA
                   A  L  S  G  L  T  S  L  Q  Q  L  S  F  G  N  Q  V  T  D  L
Lma10 inlA         GCGCTTTCAGGTTTAACTAGTCTACAGCAATTATCTTTTGGTAATCAAGTGACAGATTTA
                   A  L  S  G  L  T  S  L  Q  Q  L  S  F  G  N  Q  V  T  D  L
Lma13 inlA         GCGCTTTCAGGTTTAACTAGTCTACAGCAATTATCTTTTGGTAATCAAGTGACAGATTTA
                   A  L  S  G  L  T  S  L  Q  Q  L  S  F  G  N  Q  V  T  D  L
Lma20 inlA         GCGCTTTCAGGTTTAACTAGTCTACAGCAATTATCTTTTGGTAATCAAGTGACAGATTTA
                   A  L  S  G  L  T  S  L  Q  Q  L  S  F  G  N  Q  V  T  D  L
Lma28 inlA         GCGCTTTCAGGTTTAACTAGTCTACAGCAATTATCTTTTGGTAATCAAGTGACAGATTTA
                   A  L  S  G  L  T  S  L  Q  Q  L  S  F  G  N  Q  V  T  D  L

EGDe inlA      601 AAACCATTAGCTAATTTAACAACACTAGAACGACTAGATATTTCAAGTAATAAGGTGTCG 660
                   K  P  L  A  N  L  T  T  L  E  R  L  D  I  S  S  N  K  V  S
36-25-1 inlA       AAACCATTAGCTAATTTAACAACACTAGAACGACTAGATATTTCAAGTAATAAGGTGTCG
                   K  P  L  A  N  L  T  T  L  E  R  L  D  I  S  S  N  K  V  S
Lma10 inlA         AAACCATTAGCTAATTTAACAACACTAGAACGACTAGATATTTCAAGTAATAAGGTGTCG
                   K  P  L  A  N  L  T  T  L  E  R  L  D  I  S  S  N  K  V  S
Lma13 inlA         AAACCATTAGCTAATTTAACAACACTAGAACGACTAGATATTTCAAGTAATAAGGTGTCG
                   K  P  L  A  N  L  T  T  L  E  R  L  D  I  S  S  N  K  V  S
Lma20 inlA         AAACCATTAGCTAATTTAACAACACTAGAACGACTAGATATTTCAAGTAATAAGGTGTCG
                   K  P  L  A  N  L  T  T  L  E  R  L  D  I  S  S  N  K  V  S
Lma28 inlA         AAACCATTAGCTAATTTAACGACACTAGAACGACTAGATATTTCAAGTAATAAGGTGTCA
                   K  P  L  A  N  L  T  T  L  E  R  L  D  I  S  S  N  K  V  S

EGDe inlA      661 GATATTAGTGTTCTGGCTAAATTAACCAATTTAGAAAGTCTTATCGCTACTAACAACCAA 720
                   D  I  S  V  L  A  K  L  T  N  L  E  S  L  I  A  T  N  N  Q
36-25-1 inlA       GATATTAGTGTTCTGGCTAAATTAACCAATTTAGAAAGTCTTATCGCTACTAACAACCAA
                   D  I  S  V  L  A  K  L  T  N  L  E  S  L  I  A  T  N  N  Q
Lma10 inlA         GATATTAGTGTTCTGGCTAAATTAACCAATTTAGAAAGTCTTATCGCTACTAACAACCAA
                   D  I  S  V  L  A  K  L  T  N  L  E  S  L  I  A  T  N  N  Q
Lma13 inlA         GATATTAGTGTTCTGGCTAAATTAACCAATTTAGAAAGTCTTATCGCTACTAACAACCAA
                   D  I  S  V  L  A  K  L  T  N  L  E  S  L  I  A  T  N  N  Q
Lma20 inlA         GATATTAGTGTTCTGGCTAAATTAACCAATTTAGAAAGTCTTATCGCTACTAACAACCAA
                   D  I  S  V  L  A  K  L  T  N  L  E  S  L  I  A  T  N  N  Q
Lma28 inlA         GATATTAGTGTTCTGGCTAAATTAACCAATTTAGAAAGTCTTATCGCTACTAACAACCAA
                   D  I  S  V  L  A  K  L  T  N  L  E  S  L  I  A  T  N  N  Q
```

```
EGDe inlA       721 ATAAGTGATATAACTCCACTTGGGATTTTAACAAATTTGGACGAATTATCCTTAAATGGT 780
                    I  S  D  I  T  P  L  G  I  L  T  N  L  D  E  L  S  L  N  G
36-25-1 inlA        ATAAGTGATATAACTCCACTTGGGATTTTAACAAATTTGGACGAATTATCCTTAAATGGT
                    I  S  D  I  T  P  L  G  I  L  T  N  L  D  E  L  S  L  N  G
Lma10 inlA          ATAAGTGATATAACTCCACTTGGGATTTTAACAAATTTGGACGAATTATCCTTAAATGGT
                    I  S  D  I  T  P  L  G  I  L  T  N  L  D  E  L  S  L  N  G
Lma13 inlA          ATAAGTGATATAACTCCACTTGGGATTTTAACAAATTTGGACGAATTATCCTTAAATGGT
                    I  S  D  I  T  P  L  G  I  L  T  N  L  D  E  L  S  L  N  G
Lma20 inlA          ATAAGTGATATAACTCCACTTGGGATTTTAACAAATTTGGACGAATTATCCTTAAATGGT
                    I  S  D  I  T  P  L  G  I  L  T  N  L  D  E  L  S  L  N  G
Lma28 inlA          ATAAGTGATATAACTCCACTTGGGATTTTAACAAATTTGGACGAATTATCCTTAAATGGT
                    I  S  D  I  T  P  L  G  I  L  T  N  L  D  E  L  S  L  N  G

EGDe inlA       781 AACCAGTTAAAAGATATAGGCACATTGGCGAGTTTAACAAACCTTACAGATTTAGATTTA 840
                    N  Q  L  K  D  I  G  T  L  A  S  L  T  N  L  T  D  L  D  L
36-25-1 inlA        AACCAGTTAAAAGATATAGGCACATTGGCGAGTTTAACAAACCTTACAGATTTAGATTTA
                    N  Q  L  K  D  I  G  T  L  A  S  L  T  N  L  T  D  L  D  L
Lma10 inlA          AACCAGTTAAAAGATATAGGCACATTGGCGAGTTTAACAAACCTTACAGATTTAGATTTA
                    N  Q  L  K  D  I  G  T  L  A  S  L  T  N  L  T  D  L  D  L
Lma13 inlA          AACCAGTTAAAAGATATAGGCACATTGGCGAGTTTAACAAACCTTACAGATTTAGATTTA
                    N  Q  L  K  D  I  G  T  L  A  S  L  T  N  L  T  D  L  D  L
Lma20 inlA          AACCAGTTAAAAGATATAGGCACATTGGCGAGTTTAACAAACCTTACAGATTTAGATTTA
                    N  Q  L  K  D  I  G  T  L  A  S  L  T  N  L  T  D  L  D  L
Lma28 inlA          AACCAGTTAAAAGATATAGGCACATTGGCGAGTTTAACAAACCTTACAGATTTAGATTTA
                    N  Q  L  K  D  I  G  T  L  A  S  L  T  N  L  T  D  L  D  L

EGDe inlA       841 GCAAATAACCAAATTAGTAATCTAGCACCACTGTCGGGTCTAACAAAACTAACTGAGTTA 900
                    A  N  N  Q  I  S  N  L  A  P  L  S  G  L  T  K  L  T  E  L
36-25-1 inlA        GCAAATAACCAAATTAGTAATCTAGCACCACTGTCGGGTCTAACAAAACTAACTGAGTTA
                    A  N  N  Q  I  S  N  L  A  P  L  S  G  L  T  K  L  T  E  L
Lma10 inlA          GCAAATAACCAAATTAGTAATCTAGCACCACTGTCGGGTCTAACAAAACTAACTGAGTTA
                    A  N  N  Q  I  S  N  L  A  P  L  S  G  L  T  K  L  T  E  L
Lma13 inlA          GCAAATAACCAAATTAGTAATCTAGCACCACTGTCGGGTCTAACAAAACTAACTGAGTTA
                    A  N  N  Q  I  S  N  L  A  P  L  S  G  L  T  K  L  T  E  L
Lma20 inlA          GCAAATAACCAAATTAGTAATCTAGCACCACTGTCGGGTCTAACAAAACTAACTGAGTTA
                    A  N  N  Q  I  S  N  L  A  P  L  S  G  L  T  K  L  T  E  L
Lma28 inlA          GCAAATAACCAAATTAGTAATCTAGCACCACTGTCGGGTCTAACAAAACTAACTGAGTTA
                    A  N  N  Q  I  S  N  L  A  P  L  S  G  L  T  K  L  T  E  L

EGDe inlA       901 AAACTTGGAGCTAACCAAATAAGTAACATCAGTCCCCTAGCAGGTTTAACCGCACTCACT 960
                    K  L  G  A  N  Q  I  S  N  I  S  P  L  A  G  L  T  A  L  T
36-25-1 inlA        AAACTTGGAGCTAACCAAATAAGTAACATCAGTCCCCTAGCAGGTTTAACCGCACTCACT
                    K  L  G  A  N  Q  I  S  N  I  S  P  L  A  G  L  T  A  L  T
Lma10 inlA          AAACTTGGAGCTAACCAAATAAGTAACATCAGTCCCCTAGCAGGTTTAACCGCACTCACT
                    K  L  G  A  N  Q  I  S  N  I  S  P  L  A  G  L  T  A  L  T
Lma13 inlA          AAACTTGGAGCTAACCAAATAAGTAACATCAGTCCCCTAGCAGGTTTAACCGCACTCACT
                    K  L  G  A  N  Q  I  S  N  I  S  P  L  A  G  L  T  A  L  T
Lma20 inlA          AAACTTGGAGCTAACCAAATAAGTAACATCAGTCCCCTAGCAGGTTTAACCGCACTCACT
                    K  L  G  A  N  Q  I  S  N  I  S  P  L  A  G  L  T  A  L  T
Lma28 inlA          AAACTTGGAGCTAACCAAATAAGTAACATCAGTCCCCTAGCAGGTTTAACCGCACTCACT
                    K  L  G  A  N  Q  I  S  N  I  S  P  L  A  G  L  T  A  L  T
```

```
EGDe inlA      961 AACTTAGAGCTTAATGAAAATCAGCTGGAAGATATTAGCCCAATTTCTAACCTGAAAAAT 1020
                   N  L  E  L  N  E  N  Q  L  E  D  I  S  P  I  S  N  L  K  N
36-25-1 inlA       AACTTAGAGCTTAATGAAAATCAGCTGGAAGATATTAGCCCAATTTCTAACCTGAAAAAT
                   N  L  E  L  N  E  N  Q  L  E  D  I  S  P  I  S  N  L  K  N
Lma10 inlA         AACTTAGAGCTTAATGAAAATCAGCTGGAAGATATTAGCCCAATTTCTAACCTGAAAAAT
                   N  L  E  L  N  E  N  Q  L  E  D  I  S  P  I  S  N  L  K  N
Lma13 inlA         AACTTAGAGCTTAATGAAAATCAGCTGGAAGATATTAGCCCAATTTCTAACCTGAAAAAT
                   N  L  E  L  N  E  N  Q  L  E  D  I  S  P  I  S  N  L  K  N
Lma20 inlA         AACTTAGAGCTTAATGAAAATCAGCTGGAAGATATTAGCCCAATTTCTAACCTGAAAAAT
                   N  L  E  L  N  E  N  Q  L  E  D  I  S  P  I  S  N  L  K  N
Lma28 inlA         AACTTAGAGCTTAATGAAAATCAGCTGGAAGATATTAGCCCAATTTCTAACCTGAAAAAT
                   N  L  E  L  N  E  N  Q  L  E  D  I  S  P  I  S  N  L  K  N
```

```
EGDe inlA     1021 CTCACATATTTAACTTTGTACTTTAATAATATAAGTGATATAAGCCCAGTTTCTAGTTTA 1080
                   L  T  Y  L  T  L  Y  F  N  N  I  S  D  I  S  P  V  S  S  L
36-25-1 inlA       CTCACATATTTAACTTTGTACTTTAATAATATAAGTGATATAAGCCCAGTTTCTAGTTTA
                   L  T  Y  L  T  L  Y  F  N  N  I  S  D  I  S  P  V  S  S  L
Lma10 inlA         CTCACATATTTAACTTTGTACTTTAATAATATAAGTGATATAAGCCCAGTTTCTAGTTTA
                   L  T  Y  L  T  L  Y  F  N  N  I  S  D  I  S  P  V  S  S  L
Lma13 inlA         CTCACATATTTAACTTTGTACTTTAATAATATAAGTGATATAAGCCCAGTTTCTAGTTTA
                   L  T  Y  L  T  L  Y  F  N  N  I  S  D  I  S  P  V  S  S  L
Lma20 inlA         CTCACATATTTAACTTTGTACTTTAATAATATAAGTGATATAAGCCCAGTTTCTAGTTTA
                   L  T  Y  L  T  L  Y  F  N  N  I  S  D  I  S  P  V  S  S  L
Lma28 inlA         CTCACATATTTAACTTTGTACTTTAATAATATAAGTGATATAAGCCCAGTTTCTAGTTTA
                   L  T  Y  L  T  L  Y  F  N  N  I  S  D  I  S  P  V  S  S  L
```

```
EGDe inlA     1081 ACAAAGCTTCAAAGATTATTTTTCTATAATAACAAGGTAAGTGACGTAAGCTCACTTGCG 1140
                   T  K  L  Q  R  L  F  F  Y  N  N  K  V  S  D  V  S  S  L  A
36-25-1 inlA       ACAAAGCTTCAAAGATTATTTTTCTATAATAACAAGGTAAGTGACGTAAGCTCACTTGCG
                   T  K  L  Q  R  L  F  F  Y  N  N  K  V  S  D  V  S  S  L  A
Lma10 inlA         ACAAAGCTTCAAAGATTATTTTTCTATAATAACAAGGTAAGTGACGTAAGCTCACTTGCG
                   T  K  L  Q  R  L  F  F  Y  N  N  K  V  S  D  V  S  S  L  A
Lma13 inlA         ACAAAGCTTCAAAGATTATTTTTCTATAATAACAAGGTAAGTGACGTAAGCTCACTTGCG
                   T  K  L  Q  R  L  F  F  Y  N  N  K  V  S  D  V  S  S  L  A
Lma20 inlA         ACAAAGCTTCAAAGATTATTTTTCTATAATAACAAGGTAAGTGACGTAAGCTCACTTGCG
                   T  K  L  Q  R  L  F  F  Y  N  N  K  V  S  D  V  S  S  L  A
Lma28 inlA         ACAAAGCTTCAAAGATTATTTTTCTATAATAACAAGGTAAGTGACGTAAGCTCACTTGCG
                   T  K  L  Q  R  L  F  F  Y  N  N  K  V  S  D  V  S  S  L  A
```

```
EGDe inlA     1141 AACTTAACAAATATTAATTGGCTTTCAGCTGGGCATAACCAAATTAGCGATCTTACACCA 1200
                   N  L  T  N  I  N  W  L  S  A  G  H  N  Q  I  S  D  L  T  P
36-25-1 inlA       AACTTAACAAATATTAATTGGCTTTCAGCTGGGCATAACCAAATTAGCGATCTTACACCA
                   N  L  T  N  I  N  W  L  S  A  G  H  N  Q  I  S  D  L  T  P
Lma10 inlA         AACTTAACAAATATTAATTGGCTTTCAGCTGGGCATAACCAAATTAGCGATCTTACACCA
                   N  L  T  N  I  N  W  L  S  A  G  H  N  Q  I  S  D  L  T  P
Lma13 inlA         AACTTAACAAATATTAATTGGCTTTCAGCTGGGCATAACCAAATTAGCGATCTTACACCA
                   N  L  T  N  I  N  W  L  S  A  G  H  N  Q  I  S  D  L  T  P
Lma20 inlA         AACTTAACAAATATTAATTGGCTTTCAGCTGGGCATAACCAAATTAGCGATCTTACACCA
                   N  L  T  N  I  N  W  L  S  A  G  H  N  Q  I  S  D  L  T  P
Lma28 inlA         AACTTAACAAATATTAATTGGCTTTCAGCTGGGCATAACCAAATTAGCGATCTTACACCA
                   N  L  T  N  I  N  W  L  S  A  G  H  N  Q  I  S  D  L  T  P
```

```
EGDe inlA     1201 TTGGCTAATTTAACAAGAATCACCCAACTAGGGTTGAATGATCAAGCATGGACAAATGCA 1260
                   L  A  N  L  T  R  I  T  Q  L  G  L  N  D  Q  A  W  T  N  A
36-25-1 inlA       TTGGCTAATTTAACAAGAATCACCCAACTAGGGTTGAATGATCAAGCATGGACAAATGCA
                   L  A  N  L  T  R  I  T  Q  L  G  L  N  D  Q  A  W  T  N  A
Lma10 inlA         TTGGCTAATTTAACAAGAATCACCCAACTAGGGTTGAATGATCAAGCATGGACAAATGCA
                   L  A  N  L  T  R  I  T  Q  L  G  L  N  D  Q  A  W  T  N  A
Lma13 inlA         TTGGCTAATTTAACAAGAATCACCCAACTAGGGTTGAATGATCAAGCATGGACAAATGCA
                   L  A  N  L  T  R  I  T  Q  L  G  L  N  D  Q  A  W  T  N  A
Lma20 inlA         TTGGCTAATTTAACAAGAATCACCCAACTAGGGTTGAATGATCAAGCATGGACAAATGCA
                   L  A  N  L  T  R  I  T  Q  L  G  L  N  D  Q  A  W  T  N  A
Lma28 inlA         TTGGCTAATTTAACAAGAATCACCCAACTAGGGTTGAATGATCAAGCATGGACAAATGCA
                   L  A  N  L  T  R  I  T  Q  L  G  L  N  D  Q  A  W  T  N  A

EGDe inlA     1261 CCAGTAAACTACAAAGCAAATGTATCCATTCCAAACACGGTGAAAAATGTGACTGGCGCT 1320
                   P  V  N  Y  K  A  N  V  S  I  P  N  T  V  K  N  V  T  G  A
36-25-1 inlA       CCAGTAAACTACAAAGCAAATGTATCCATTCCAAACACGGTGAAAAATGTGACTGGCGCT
                   P  V  N  Y  K  A  N  V  S  I  P  N  T  V  K  N  V  T  G  A
Lma10 inlA         CCAGTAAACTACAAAGCAAATGTATCCATTCCAAACACGGTGAAAAATGTGACTGGCGCT
                   P  V  N  Y  K  A  N  V  S  I  P  N  T  V  K  N  V  T  G  A
Lma13 inlA         CCAGTAAACTACAAAGCAAATGTATCCATTCCAAACACGGTGAAAAATGTGACTGGCGCT
                   P  V  N  Y  K  A  N  V  S  I  P  N  T  V  K  N  V  T  G  A
Lma20 inlA         CCAGTAAACTACAAAGCAAATGTATCCATTCCAAACACGGTGAAAAATGTGACTGGCGCT
                   P  V  N  Y  K  A  N  V  S  I  P  N  T  V  K  N  V  T  G  A
Lma28 inlA         CCAGTAAACTACAAAGCAAATGTATCCATTCCAAACACGGTGAAAAATGTGACTGGCGCT
                   P  V  N  Y  K  A  N  V  S  I  P  N  T  V  K  N  V  T  G  A

EGDe inlA     1321 TTAATTGCACCAGCTACTATTAGCGATGGCGGTAGTTACACAGAGCCTGATATAACATGG 1380
                   L  I  A  P  A  T  I  S  D  G  G  S  Y  T  E  P  D  I  T  W
36-25-1 inlA       TTAATTGCACCAGCTACTATTAGCGATGGCGGTAGTTACACAGAGCCTGATATAACATGG
                   L  I  A  P  A  T  I  S  D  G  G  S  Y  T  E  P  D  I  T  W
Lma10 inlA         TTAATTGCACCAGCTACTATTAGCGATGGCGGTAGTTACACAGAGCCTGATATAACATGG
                   L  I  A  P  A  T  I  S  D  G  G  S  Y  T  E  P  D  I  T  W
Lma13 inlA         TTAATTGCACCAGCTACTATTAGCGATGGCGGTAGTTACACAGAGCCTGATATAACATGG
                   L  I  A  P  A  T  I  S  D  G  G  S  Y  T  E  P  D  I  T  W
Lma20 inlA         TTAATTGCACCAGCTACTATTAGCGATGGCGGTAGTTACACAGAGCCTGATATAACATGG
                   L  I  A  P  A  T  I  S  D  G  G  S  Y  T  E  P  D  I  T  W
Lma28 inlA         TTGATTGCACCAGCTACTATTAGCGATGGTGGTAGTTATGCAGAACCTGATATAACATGG
                   L  I  A  P  A  T  I  S  D  G  G  S  Y  A  E  P  D  I  T  W

EGDe inlA     1381 AACTTACCTAGTTATACAAATGAAGTAAGCTATACCTTTAGCCAACCTGTCACTATTGGA 1440
                   N  L  P  S  Y  T  N  E  V  S  Y  T  F  S  Q  P  V  T  I  G
36-25-1 inlA       AACTTACCTAGTTATACAAATGAAGTAAGCTATACCTTTAGCCAACCTGTCACTATTGGA
                   N  L  P  S  Y  T  N  E  V  S  Y  T  F  S  Q  P  V  T  I  G
Lma10 inlA         AACTTACCTAGTTATACAAATGAAGTAAGCTATACCTTTAGCCAACCTGTCACTATTGGA
                   N  L  P  S  Y  T  N  E  V  S  Y  T  F  S  Q  P  V  T  I  G
Lma13 inlA         AACTTACCTAGTTATACAAATGAAGTAAGCTATACCTTTAGCCAACCTGTCACTATTGGA
                   N  L  P  S  Y  T  N  E  V  S  Y  T  F  S  Q  P  V  T  I  G
Lma20 inlA         AACTTACCTAGTTATACAAATGAAGTAAGCTATACCTTTAGCCAACCTGTCACTATTGGA
                   N  L  P  S  Y  T  N  E  V  S  Y  T  F  S  Q  P  V  T  I  G
Lma28 inlA         AACTTACCTAGTTATACGAATGAAGTAAGTTATACCTTTAACCAATCTGTCACCATTGGA
                   N  L  P  S  Y  T  N  E  V  S  Y  T  F  N  Q  S  V  T  I  G
```

```
EGDe inlA      1441 AAAGGAACGACAACATTTAGTGGAACCGTGACGCAGCCACTTAAGGCAATTTTTAATGTT 1500
                    K G T T T F S G T V T Q P L K A I F N V
36-25-1 inlA        AAAGGAACGACAACATTTAGTGGAACCGTGACGCAGCCACTTAAGGCAATTTTTAATGTT
                    K G T T T F S G T V T Q P L K A I F N V
Lma10 inlA          AAAGGAACGACAACATTTAGTGGAACCGTGACGCAGCCACTTAAGGCAATTTTTAATGTT
                    K G T T T F S G T V T Q P L K A I F N V
Lma13 inlA          AAAGGAACGACAACATTTAGTGGAACCGTGACGCAGCCACTTAAGGCAATTTTTAATGTT
                    K G T T T F S G T V T Q P L K A I F N V
Lma20 inlA          AAAGGAACGACAACATTTAGTGGAACCGTGACGCAGCCACTTAAGGCAATTTTTAATGTT
                    K G T T T F S G T V T Q P L K A I F N V
Lma28 inlA          AAAGGAACGACAACATTTAGTGGAACTGTGACGTAGCCACTTAAGGCAATTTTTAATGCT
                    K G T T T F S G T V T * P L K A I F N A

EGDe inlA      1501 AAGTTTCATGTGGACGGCAAAGAAACAACCAAAGAAGTGGAAGCTGGGAATTTATTGACT 1560
                    K F H V D G K E T T K E V E A G N L L T
36-25-1 inlA        AAGTTTCATGTGGACGGCAAAGAAACAACCAAAGAAGTGGAAGCTGGGAATTTATTGACT
                    K F H V D G K E T T K E V E A G N L L T
Lma10 inlA          AAGTTTCATGTGGACGGCAAAGAAACAACCAAAGAAGTGGAAGCTGGGAATTTATTGACT
                    K F H V D G K E T T K E V E A G N L L T
Lma13 inlA          AAGTTTCATGTGGACGGCAAAGAAACAACCAAAGAAGTGGAAGCTGGGAATTTATTGACT
                    K F H V D G K E T T K E V E A G N L L T
Lma20 inlA          AAGTTTCATGTGGACGGCAAAGAAACAACCAAAGAAGTGGAAGCTGGGAATTTATTGACT
                    K F H V D G K E T T K E V E A G N L L T
Lma28 inlA          AAGTTTCATGTGGACGGCAAAGAAACAACCAAAGAAGTGGAAGCTGGGAATTTATTGACT
                    K F H V D G K E T T K E V E A G N L L T

EGDe inlA      1561 GAACCAGCTAAGCCCGTAAAAGAAGGTCACACATTTGTTGGTTGGTTTGATGCCCAAACA 1620
                    E P A K P V K E G H T F V G W F D A Q T
36-25-1 inlA        GAACCAGCTAAGCCCGTATAAGAAGGTCACACATTTGTTGGTTGGTTTGATGCCCAAACA
                    E P A K P V * E G H T F V G W F D A Q T
Lma10 inlA          GAACCAGCTAAGCCCGTAAAAGAAGGTCACACATTTGTTGGTTGGTTTGATGCCCAAACA
                    E P A K P V K E G H T F V G W F D A Q T
Lma13 inlA          GAACCAGCTAAGCCCGTAAAAGAAGGTCACACATTTGTTGGTTGGTTTGATGCCCAAACA
                    E P A K P V K E G H T F V G W F D A Q T
Lma20 inlA          GAACCAGCTAAGCCCGTAAAAGAAGGTCACACATTTGTTGGTTGGTTTGATGCCCAAACA
                    E P A K P V K E G H T F V G W F D A Q T
Lma28 inlA          GAACCAGCTAAGCCTGTAAAAGAAGGTTACACATTTATTGGGTGGTTTGATGCCAAAACA
                    E P A K P V K E G Y T F I G W F D A K T

EGDe inlA      1621 GGCGGAACTAAATGGAATTTCAGTACGGATAAAATGCCGACAAATGACATCAATTTATAT 1680
                    G G T K W N F S T D K M P T N D I N L Y
36-25-1 inlA        GGCGGAACTAAATGGAATTTCAGTACGGATAAAATGCCGACAAATGACATCAATTTATAT
                    G G T K W N F S T D K M P T N D I N L Y
Lma10 inlA          GGCGGAACTAAATGGAATTTCAGTACGGATAAAATGCCGACAAATGACATCAATTTATAT
                    G G T K W N F S T D K M P T N D I N L Y
Lma13 inlA          GGCGGAACTAAATGGAATTTCAGTACGGATAAAATGCCGACAAATGACATCAATTTATAT
                    G G T K W N F S T D K M P T N D I N L Y
Lma20 inlA          GGCGGAACTAAATGGAATTTCAGTACGGATAAAATGCCGACAAATGACATCAATTTATAT
                    G G T K W N F S T D K M P T N D I N L Y
Lma28 inlA          GGTGGAACTAAATGGAATTTCAGTACGGATAAAATGCCGACAAATGACATCGATTTATAT
                    G G T K W N F S T D K M P T N D I D L Y
```

```
EGDe inlA      1681 GCACAATTTAGTATTAACAGCTACACAGCAACCTTTGATAATGACGGTGTAACAACATCT 1740
                    A  Q  F  S  I  N  S  Y  T  A  T  F  D  N  D  G  V  T  T  S
36-25-1 inlA        GCACAATTTAGTATTAACAGCTACACAGCAACCTTTGATAATGACGGTGTAACAACATCT
                    A  Q  F  S  I  N  S  Y  T  A  T  F  D  N  D  G  V  T  T  S
Lma10 inlA          GCACAATTTAGTATTAACAGCTACACAGCAACCTTTGATAATGACGGTGTAACAACATCT
                    A  Q  F  S  I  N  S  Y  T  A  T  F  D  N  D  G  V  T  T  S
Lma13 inlA          GCACAATTTAGTATTAACAGCTACACAGCAACCTTTGATAATGACGGTGTAACAACATCT
                    A  Q  F  S  I  N  S  Y  T  A  T  F  D  N  D  G  V  T  T  S
Lma20 inlA          GCACAATTTAGTATTAACAGCTACACAGCAACCTTTGATAATGACGGTGTAACAACATCT
                    A  Q  F  S  I  N  S  Y  T  A  T  F  D  N  D  G  V  T  T  S
Lma28 inlA          GCGCAATTTAGTATTAACAGCTACACAGCAACCTTGGATAATGACGGTGTAACAACATCT
                    A  Q  F  S  I  N  S  Y  T  A  T  L  D  N  D  G  V  T  T  S

EGDe inlA      1741 CAAACAGTAGATTATCAAGGCTTGTTACAAGAACCTACGGCACCAACAAAAGAAGGTTAT 1800
                    Q  T  V  D  Y  Q  G  L  L  Q  E  P  T  A  P  T  K  E  G  Y
36-25-1 inlA        CAAACAGTAGATTATCAAGGCTTGTTACAAGAACCTACGGCACCAACAAAAGAAGGTTAT
                    Q  T  V  D  Y  Q  G  L  L  Q  E  P  T  A  P  T  K  E  G  Y
Lma10 inlA          CAAACAGTAGATTATCAAGGCTTGTTACAAGAACCTACGGCACCAACAAAAGAAGGTTAT
                    Q  T  V  D  Y  Q  G  L  L  Q  E  P  T  A  P  T  K  E  G  Y
Lma13 inlA          CAAACAGTAGATTATCAAGGCTTGTTACAAGAACCTACGGCACCAACAAAAGAAGGTTAT
                    Q  T  V  D  Y  Q  G  L  L  Q  E  P  T  A  P  T  K  E  G  Y
Lma20 inlA          CAAACAGTAGATTATCAAGGCTTGTTACAAGAACCTACGGCACCAACAAAAGAAGGTTAT
                    Q  T  V  D  Y  Q  G  L  L  Q  E  P  T  A  P  T  K  E  G  Y
Lma28 inlA          CAAACAGTAGATTATCAAGGCTTGCTACAAGAACCTACGGCACCAACAAAAGAAGGTTAT
                    Q  T  V  D  Y  Q  G  L  L  Q  E  P  T  A  P  T  K  E  G  Y

EGDe inlA      1801 ACTTTTAAAGGCTGGTATGACGCAAAAACTGGTGGTGACAAGTGGGATTTCGCAACTAGC 1860
                    T  F  K  G  W  Y  D  A  K  T  G  G  D  K  W  D  F  A  T  S
36-25-1 inlA        ACTTTTAAAGGCTGGTATGACGCAAAAACTGGTGGTGACAAGTGGGATTTCGCAACTAGC
                    T  F  K  G  W  Y  D  A  K  T  G  G  D  K  W  D  F  A  T  S
Lma10 inlA          ACTTTTAAAGGCTGGTATGACGCAAAAACTGGTGGTGACAAGTGGGATTTCGCAACTAGC
                    T  F  K  G  W  Y  D  A  K  T  G  G  D  K  W  D  F  A  T  S
Lma13 inlA          ACTTTTAAAGGCTGGTATGACGCAAAAACTGGTGGTGACAAGTGGGATTTCGCAACTAGC
                    T  F  K  G  W  Y  D  A  K  T  G  G  D  K  W  D  F  A  T  S
Lma20 inlA          ACTTTTAAAGGCTGGTATGACGCAAAAACTGGTGGTGACAAGTGGGATTTCGCAACTAGC
                    T  F  K  G  W  Y  D  A  K  T  G  G  D  K  W  D  F  A  T  S
Lma28 inlA          ACTTTTAAAGGCTGGTATGACGCAAAAACTGGCGGTGACAAGTGGGATTTTGCAACTAGT
                    T  F  K  G  W  Y  D  A  K  T  G  G  D  K  W  D  F  A  T  S

EGDe inlA      1861 AAAATGCCTGCTAAAAACATCACCTTATATGCCCAATATAGCGCCAATAGCTATACAGCA 1920
                    K  M  P  A  K  N  I  T  L  Y  A  Q  Y  S  A  N  S  Y  T  A
36-25-1 inlA        AAAATGCCTGCTAAAAACATCACCTTATATGCCCAATATAGCGCCAATAGCTATACAGCA
                    K  M  P  A  K  N  I  T  L  Y  A  Q  Y  S  A  N  S  Y  T  A
Lma10 inlA          AAAATGCCTGCTAAAAACATCACCTTATATGCCCAATATAGCGCCAATAGCTATACAGCA
                    K  M  P  A  K  N  I  T  L  Y  A  Q  Y  S  A  N  S  Y  T  A
Lma13 inlA          AAAATGCCTGCTAAAAACATCACCTTATATGCCCAATATAGCGCCAATAGCTATACAGCA
                    K  M  P  A  K  N  I  T  L  Y  A  Q  Y  S  A  N  S  Y  T  A
Lma20 inlA          AAAATGCCTGCTAAAAACATCACCTTATATGCCCAATATAGCGCCAATAGCTATACAGCA
                    K  M  P  A  K  N  I  T  L  Y  A  Q  Y  S  A  N  S  Y  T  A
Lma28 inlA          AAGATGCCTGCTAAAAACATCACCTTATATGCTCAATATAGCGCCAATAGCTATACAGCA
                    K  M  P  A  K  N  I  T  L  Y  A  Q  Y  S  A  N  S  Y  T  A
```

```
EGDe inlA     1921 ACGTTTGATGTTGATGGAAAATCAACGACTCAAGCAGTAGACTATCAAGGACTTCTAAAA 1980
                   T  F  D  V  D  G  K  S  T  T  Q  A  V  D  Y  Q  G  L  L  K
36-25-1 inlA       ACGTTTGATGTTGATGGAAAATCAACGACTCAAGCAGTAGACTATCAAGGACTTCTAAAA
                   T  F  D  V  D  G  K  S  T  T  Q  A  V  D  Y  Q  G  L  L  K
Lma10 inlA         ACGTTTGATGTTGATGGAAAATCAACGACTCAAGCAGTAGACTATCAAGGACTTCTAAAA
                   T  F  D  V  D  G  K  S  T  T  Q  A  V  D  Y  Q  G  L  L  K
Lma13 inlA         ACGTTTGATGTTGATGGAAAATCAACGACTCAAGCAGTAGACTATCAAGGACTTCTAAAA
                   T  F  D  V  D  G  K  S  T  T  Q  A  V  D  Y  Q  G  L  L  K
Lma20 inlA         ACGTTTGATGTTGATGGAAAATCAACGACTCAAGCAGTAGACTATCAAGGACTTCTAAAA
                   T  F  D  V  D  G  K  S  T  T  Q  A  V  D  Y  Q  G  L  L  K
Lma28 inlA         ACCTTTGATGTTGATGGAAAAACAACGACTCAAGCAGTAGACTATCAAGGACTTCTAAAA
                   T  F  D  V  D  G  K  T  T  T  Q  A  V  D  Y  Q  G  L  L  K

EGDe inlA     1981 GAACCAAAGGCACCAACGAAAGCCGGATATACTTTCAAAGGCTGGTATGACGAAAAAACA 2040
                   E  P  K  A  P  T  K  A  G  Y  T  F  K  G  W  Y  D  E  K  T
36-25-1 inlA       GAACCAAAGGCACCAACGAAAGCCGGATATACTTTCAAAGGCTGGTATGACGAAAAAACA
                   E  P  K  A  P  T  K  A  G  Y  T  F  K  G  W  Y  D  E  K  T
Lma10 inlA         GAACCAAAGGCACCAACGAAAGCCGGATATACTTTCAAAGGCTGGTATGACGAAAAAACA
                   E  P  K  A  P  T  K  A  G  Y  T  F  K  G  W  Y  D  E  K  T
Lma13 inlA         GAACCAAAGGCACCAACGAAAGCCGGATATACTTTCAAAGGCTGGTATGACGAAAAAACA
                   E  P  K  A  P  T  K  A  G  Y  T  F  K  G  W  Y  D  E  K  T
Lma20 inlA         GAACCAAAGGCACCAACGAAAGCCGGATATACTTTCAAAGGCTGGTATGACGAAAAAACA
                   E  P  K  A  P  T  K  A  G  Y  T  F  K  G  W  Y  D  E  K  T
Lma28 inlA         GAACCAAAAACGCCAACAAAAGCCGGATATACTTTCAAAGGTTGGTATGACGAAAAAACA
                   E  P  K  T  P  T  K  A  G  Y  T  F  K  G  W  Y  D  E  K  T

EGDe inlA     2041 GATGGGAAAAAATGGGATTTTGCGACGGATAAAATGCCAGCAAATGACATTACGCTGTAC 2100
                   D  G  K  K  W  D  F  A  T  D  K  M  P  A  N  D  I  T  L  Y
36-25-1 inlA       GATGGGAAAAAATGGGATTTTGCGACGGATAAAATGCCAGCAAATGACATTACGCTGTAC
                   D  G  K  K  W  D  F  A  T  D  K  M  P  A  N  D  I  T  L  Y
Lma10 inlA         GATGGGAAAAAATGGGATTTTGCGACGGATAAAATGCCAGCAAATGACATTACGCTGTAG
                   D  G  K  K  W  D  F  A  T  D  K  M  P  A  N  D  I  T  L  *
Lma13 inlA         GATGGGAAAAAATGGGATTTTGCGACGGATAAAATGCCAGCAAATGACATTACGCTGTAG
                   D  G  K  K  W  D  F  A  T  D  K  M  P  A  N  D  I  T  L  *
Lma20 inlA         GATGGGAAAAAATGGGATTTTGCGACGGATAAAATGCCAGCAAATGACATTACGCTGTAG
                   D  G  K  K  W  D  F  A  T  D  K  M  P  A  N  D  I  T  L  *
Lma28 inlA         GATGGTAAAAAATGGGATTTTGCGACAGATAAAATGCCAGCAAATGATATTACGCTGTAC
                   D  G  K  K  W  D  F  A  T  D  K  M  P  A  N  D  I  T  L  Y

EGDe inlA     2101 GCTCAATTTACGAAAAATCCTGTGGCACCACCAACAACTGGAGGGAACACACCGCCTACA 2160
                   A  Q  F  T  K  N  P  V  A  P  P  T  T  G  G  N  T  P  P  T
36-25-1 inlA       GCTCAATTTACGAAAAATCCTGTGGCACCACCAACAACTGGAGGGAACACACCGCCTACA
                   A  Q  F  T  K  N  P  V  A  P  P  T  T  G  G  N  T  P  P  T
Lma10 inlA         GCTCAATTTACGAAAAATCCTGTGGCACCACCAACAACTGGAGGGAACACACCGCCTACA
                   A  Q  F  T  K  N  P  V  A  P  P  T  T  G  G  N  T  P  P  T
Lma13 inlA         GCTCAATTTACGAAAAATCCTGTGGCACCACCAACAACTGGAGGGAACACACCGCCTACA
                   A  Q  F  T  K  N  P  V  A  P  P  T  T  G  G  N  T  P  P  T
Lma20 inlA         GCTCAATTTACGAAAAATCCTGTGGCACCACCAACAACTGGAGGGAACACACCGCCTACA
                   A  Q  F  T  K  N  P  V  A  P  P  T  T  G  G  N  T  P  P  T
Lma28 inlA         GCTCAATTCACGAAAAATCCTGTGGCACCACCAACAACTGGAGGGAACACGCCGCCGACT
                   A  Q  F  T  K  N  P  V  A  P  P  T  T  G  G  N  T  P  P  T
```

```
EGDe inlA     2161 ACAAATAACGGCGGGAATACTACACCACCTTCCGCAAATATACCTGGAAGCGACACATCT 2220
                   T  N  N  G  G  N  T  T  P  P  S  A  N  I  P  G  S  D  T  S
36-25-1 inlA       ACAAATAACGGCGGGAATACTACACCACCTTCCGCAAATATACCTGGAAGCGACACATCT
                   T  N  N  G  G  N  T  T  P  P  S  A  N  I  P  G  S  D  T  S
Lma10 inlA         ACAAATAACGGCGGGAATACTACACCACCTTCCGCAAATATACCTGGAAGCGACACATCT
                   T  N  N  G  G  N  T  T  P  P  S  A  N  I  P  G  S  D  T  S
Lma13 inlA         ACAAATAACGGCGGGAATACTACACCACCTTCCGCAAATATACCTGGAAGCGACACATCT
                   T  N  N  G  G  N  T  T  P  P  S  A  N  I  P  G  S  D  T  S
Lma20 inlA         ACAAATAACGGCGGGAATACTACACCACCTTCCGCAAATATACCTGGAAGCGACACATCT
                   T  N  N  G  G  N  T  T  P  P  S  A  N  I  P  G  S  D  T  S
Lma28 inlA         ACAAATAACGGAGGGAATACTACACCACCTTCCGCAAATATACCTGGAAGCGACACATCT
                   T  N  N  G  G  N  T  T  P  P  S  A  N  I  P  G  S  D  T  S

EGDe inlA     2221 AACACATCAACTGGGAATTCAGCCAGCACAACAAGTACAATGAACGCTTATGACCCTTAT 2280
                   N  T  S  T  G  N  S  A  S  T  T  S  T  M  N  A  Y  D  P  Y
36-25-1 inlA       AACACATCAACTGGGAATTCAGCCAGCACAACAAGTACAATGAACGCTTATGACCCTTAT
                   N  T  S  T  G  N  S  A  S  T  T  S  T  M  N  A  Y  D  P  Y
Lma10 inlA         AACACATCAACTGGGAATTCAGCCAGCACAACAAGTACAATGAACGCTTATGACCCTTAT
                   N  T  S  T  G  N  S  A  S  T  T  S  T  M  N  A  Y  D  P  Y
Lma13 inlA         AACACATCAACTGGGAATTCAGCCAGCACAACAAGTACAATGAACGCTTATGACCCTTAT
                   N  T  S  T  G  N  S  A  S  T  T  S  T  M  N  A  Y  D  P  Y
Lma20 inlA         AACACATCAACTGGGAATTCAGCCAGCACAACAAGTACAATGAACGCTTATGACCCTTAT
                   N  T  S  T  G  N  S  A  S  T  T  S  T  M  N  A  Y  D  P  Y
Lma28 inlA         AACACATCAACTGGGAATTCAGCCAGCACAACAAGTACAATGAACGCTTATGACCCTTAT
                   N  T  S  T  G  N  S  A  S  T  T  S  T  M  N  A  Y  D  P  Y

EGDe inlA     2281 AATTCAAAAGAAGCTTCACTCCCTACAACTGGCGATAGCGATAATGCGCTCTACCTTTTG 2340
                   N  S  K  E  A  S  L  P  T  T  G  D  S  D  N  A  L  Y  L  L
36-25-1 inlA       AATTCAAAAGAAGCTTCACTCCCTACAACTGGCGATAGCGATAATGCGCTCTACCTTTTG
                   N  S  K  E  A  S  L  P  T  T  G  D  S  D  N  A  L  Y  L  L
Lma10 inlA         AATTCAAAAGAAGCTTCACTCCCTACAACTGGCGATAGCGATAATGCGCTCTACCTTTTG
                   N  S  K  E  A  S  L  P  T  T  G  D  S  D  N  A  L  Y  L  L
Lma13 inlA         AATTCAAAAGAAGCTTCACTCCCTACAACTGGCGATAGCGATAATGCGCTCTACCTTTTG
                   N  S  K  E  A  S  L  P  T  T  G  D  S  D  N  A  L  Y  L  L
Lma20 inlA         AATTCAAAAGAAGCTTCACTCCCTACAACTGGCGATAGCGATAATGCGCTCTACCTTTTG
                   N  S  K  E  A  S  L  P  T  T  G  D  S  D  N  A  L  Y  L  L
Lma28 inlA         AATTCAAAAGAAGCTTCACTCCCTACAACTGGCGATAGCGATAATGCGCTCTACCTTTTG
                   N  S  K  E  A  S  L  P  T  T  G  D  S  D  N  A  L  Y  L  L

EGDe inlA     2341 TTAGGGTTATTAGCAGTAGGAACTGCAATGGCTCTTACTAAAAAAGCACGTGCTAGTAAA 2400
                   L  G  L  L  A  V  G  T  A  M  A  L  T  K  K  A  R  A  S  K
36-25-1 inlA       TTAGGGTTATTAGCAGTAGGAACTGCAATGGCTCTTACTAAAAAAGCACGTGCTAGTAAA
                   L  G  L  L  A  V  G  T  A  M  A  L  T  K  K  A  R  A  S  K
Lma10 inlA         TTAGGGTTATTAGCAGTAGGAACTGCAATGGCTCTTACTAAAAAAGCACGTGCTAGTAAA
                   L  G  L  L  A  V  G  T  A  M  A  L  T  K  K  A  R  A  S  K
Lma13 inlA         TTAGGGTTATTAGCAGTAGGAACTGCAATGGCTCTTACTAAAAAAGCACGTGCTAGTAAA
                   L  G  L  L  A  V  G  T  A  M  A  L  T  K  K  A  R  A  S  K
Lma20 inlA         TTAGGGTTATTAGCAGTAGGAACTGCAATGGCTCTTACTAAAAAAGCACGTGCTAGTAAA
                   L  G  L  L  A  V  G  T  A  M  A  L  T  K  K  A  R  A  S  K
Lma28 inlA         TTAGGGTTATTAGCAGTAGGAACTGCAATGGCTCTTACTAAAAAAGCACGTGCTAGTAAA
                   L  G  L  L  A  V  G  T  A  M  A  L  T  K  K  A  R  A  S  K
```

EGDe inlA	2401	TAG<br>*
36-25-1 inlA		TAG<br>*
Lma10 inlA		TAG<br>*
Lma13 inlA		TAG<br>*
Lma20 inlA		TAG<br>*
Lma28 inlA		TAG<br>*
